# Cross-sectional study of asymptomatic malaria and seroepidemiological surveillance of seven districts in Gia Lai province, Vietnam

**DOI:** 10.1186/s12936-022-04060-6

**Published:** 2022-02-08

**Authors:** Nguyen Ngoc San, Nguyen Xuan Kien, Nguyen Duc Manh, Nguyen Van Thanh, Marina Chavchich, Nguyen Thi Huong Binh, Tran Khanh Long, Kimberly A. Edgel, Eduard Rovira-Vallbona, Michael D. Edstein, Nicholas J. Martin

**Affiliations:** 1 Hanoi Medical University, Hanoi, Vietnam; 2 Vietnam People’s Army Military Medical Department, Hanoi, Vietnam; 3 Vietnam People’s Army Military Institute of Preventive Medicine, Hanoi, Vietnam; 4 Australian Defence Force Malaria and Infectious Disease Institute, Brisbane, Australia; 5 National Institute of Malariology Parasitology and Entomology, Hanoi, Vietnam; 6 Vysnova Partners, Bethesda, MD USA; 7 U.S. Naval Medical Research Unit TWO, Singapore, Singapore

**Keywords:** Asymptomatic malaria, *Plasmodium falciparum*, *Plasmodium vivax*, Gia Lai province, Vietnam, PCR, Serology, Drug resistance

## Abstract

**Background:**

Malaria elimination by 2030 is an aim of many countries in the Greater Mekong Sub-region, including Vietnam. However, to achieve this goal and accelerate towards malaria elimination, countries need to determine the extent and prevalence of asymptomatic malaria as a potential reservoir for malaria transmission and the intensity of malaria transmission. The purpose of this study was to determine the prevalence of asymptomatic malaria and seropositivity rate in several districts of Gia Lai province in the Central Highlands of Vietnam.

**Methods:**

A cross-sectional survey of asymptomatic malaria and serological testing was conducted in 3283 people living at 14 communes across seven districts in Gia Lai province in December 2016 to January 2017. Finger prick capillary blood samples were tested for malaria using rapid diagnostic testing and polymerase chain reaction (PCR), as well as detecting antibodies against 3 *Plasmodium falciparum* and 4 *Plasmodium vivax* antigens by indirect enzyme-linked immunosorbent assay (ELISA). Age-seroprevalence curves were fitted using reverse catalytic models with maximum likelihood.

**Results:**

The study population was predominantly male (65.9%, 2165/3283), adults (88.7%, 2911/3283) and of a minority ethnicity (72.2%, 2371/3283), with most participants being farmers and outdoor government workers (90.2%, 2960/3283). Using a small volume of blood (≈ 10 µL) the PCR assay revealed that 1.74% (57/3283) of the participants had asymptomatic malaria (*P. falciparum* 1.07%, *P. vivax* 0.40%, *Plasmodium malariae* 0.15% and mixed infections 0.12%). In contrast, the annual malaria prevalence rates for clinical malaria in the communities where the participants lived were 0.12% (108/90,395) in 2016 and 0.22% (201/93,184) in 2017. Seropositivity for at least one *P. falciparum* or one *P. vivax* antigen was 38.5% (1257/3262) and 31.1% (1022/3282), respectively. Age-dependent trends in the proportion of seropositive individuals in five of the districts discriminated the three districts with sustained low malaria prevalence from the two districts with higher transmission.

**Conclusions:**

Asymptomatic *Plasmodium* carriers were found to be substantially more prevalent than clinical cases in seven districts of Gia Lai province, and a third of the population had serological evidence of previous malaria exposure. The findings add knowledge on the extent of asymptomatic malaria and transmission for developing malaria elimination strategies for Vietnam.

**Supplementary Information:**

The online version contains supplementary material available at 10.1186/s12936-022-04060-6.

## Background

Despite continued decreases in the number of malaria cases outside of Africa, there are still more than 200,000 cases reported annually across Asia and the Pacific, primarily in 11 endemic countries (Cambodia, China, the Lao People's Democratic Republic, Myanmar, Malaysia, Papua New Guinea, the Philippines, the Republic of Korea, Solomon Islands, Vanuatu and Vietnam) [1]. Countries across the Greater Mekong Subregion (GMS) have pledged to eliminate all *Plasmodium* species of malaria by 2030 [2]. There is lasting value to eliminating malaria as nearly all countries that have achieved malaria elimination have been able to maintain this status despite the presence of competent vectors, imported cases and proximity to malaria endemic countries [3]. Additionally, malaria elimination is critical to combating drug resistant malaria present across the GMS [4].

Significant reductions in malaria mortality and morbidity were observed in Vietnam between 1991 and 2013 with continued decreases reported through 2015 [5]. However, the Vietnam National Institute of Malariology, Parasitology and Entomology (NIMPE) reported an increase in the overall number of malaria cases during 2017–2018. Challenges complicating elimination plans are: (1) the presence of *P. falciparum* parasites resistant to the current artemisinin-based first-line treatments described in six provinces (Quang Nam, Gia Lai, Dak Nong, Khanh Hòa, Ninh Thuan and Binh Phuoc) [4], (2) identification and targeting of the sub-clinical infectious reservoir, and (3) the elimination of infections caused by malaria species other than *P. falciparum*. While *P. falciparum* is the focus of current elimination efforts, malaria caused by *Plasmodium vivax*, *Plasmodium malariae, Plasmodium ovale* and *Plasmodium knowlesi* may require different strategies due to either dormant liver stages (hypnozoites), importation from areas outside of Vietnam or presence of animal reservoirs [5–7].

It is widely accepted that asymptomatic malaria parasite carriers present a unique challenge for elimination programmes as they provide a transmission reservoir capable of sustaining malaria endemicity [8–10]. As Vietnam and other countries approach zero cases of malaria, a pre-requisite for World Health Organization (WHO) designation of malaria elimination, areas of residual transmission will become increasingly important to identify and treat. National Malaria Control Programmes (NMCPs) still rely almost exclusively on blood film microscopy or rapid diagnostic tests (RDTs) for the routine detection of malaria and case investigations at communal health stations and for large scale malaria surveys. Although these tools are relatively inexpensive and widely available, they lack the sensitivity to detect infections in individuals who are asymptomatic and/or carry sub-microscopic parasite densities. These infections need to be identified by more sensitive molecular tools, such as polymerase chain reaction (PCR), to aid NMCPs develop elimination strategies and target resources to areas of residual malaria transmission.

As transmission decreases, parasitological surveys designed to identify ongoing infections will require large sample sizes, becoming logistically and economically unfeasible for NMCPs. Other malaria transmission metrics such as entomological inoculation rate also have inherent limitations in low transmission areas due to very low numbers of infected mosquitoes [11–13]. Alternatively, serological markers of parasite exposure have been proposed as a potentially cost-effective proxy for malaria transmission intensity in low endemic areas [10, 11, 14, 15]. Seropositivity to parasite antigens in a specific population is assumed to represent cumulative exposure to past malaria infections thus reducing the probability of missing exposure events, including asymptomatic infections. Moreover, by analysing age-seroprevalence patterns it is possible to identify historical trends in transmission intensity in a specific population group and how these trends differ between communities [11, 16, 17]. Such serological tools are of special interest in pre-elimination scenarios existing in Vietnam, with marked spatial transmission heterogeneity (*i.e.* hotspots of transmission surrounded by areas of no transmission), where geographical stratification in transmission levels becomes essential to target NMCP interventions efficiently [18].

The primary objective of this cross-sectional study was to characterize the parasite reservoir of *P. falciparum, P. vivax, P. ovale, P. malariae* and *P. knowlesi* in an asymptomatic population working and residing in Gia Lai province, Vietnam. Three endpoints were assessed to characterize malaria burden in the study population: (1) prevalence of parasite positive individuals determined by PCR, (2) prevalence of individuals with a history of malaria exposure measured by detection of antibodies to *P. falciparum* or *P. vivax,* and (3) proportion of *P. falciparum* parasites with molecular markers associated with drug resistance.

## Methods

### Study location

**Fig. 1:**
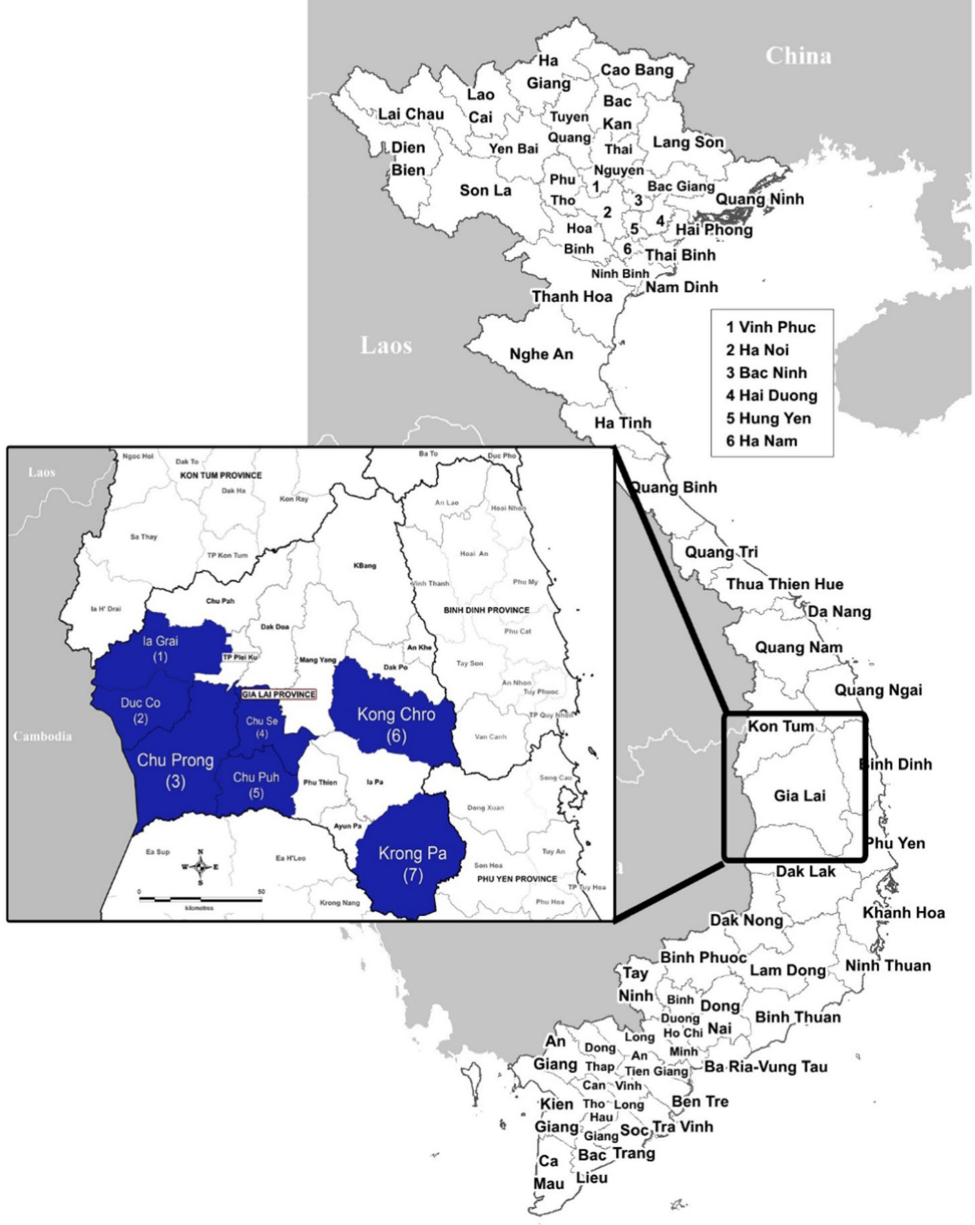
Map of Gia Lai province, Vietnam. The study districts highlighted are in blue: Ia Grai (1), Duc Co (2), Chu Prong (3), Chu Se (4), Chu Puh (5), Kon Chro (6) and Krong Pa (7)

The study was conducted between December 2016 and January 2017 at located in seven different districts of Gia Lai province. Gia Lai is located in the Central Highlands of Vietnam on the border with Cambodia, has a population of approximately 1.3 million people residing in an area of 15,500 km^2^ distributed in 17 districts and city divisions (Fig. 1). Malaria transmission is perennial, typically rising after the start of the rains in May and peaking between September and December, with the lowest incidence from February to April. Delayed *P. falciparum* malaria clearance following artemisinin-based combination therapy (*i.e.* dihydroartemisinin-piperaquine, DHA-PPQ) was reported in Gia Lai province in 2010 and 2017 [19, 20].

Cross-sectional malaria surveys were done in 14 military-civilian health facilities across the seven districts: Chu Prong (Ia Mo and Ia Puch communes), Chu Puh (Ia Hla, Ia Le and Ia Phang communes), Chu Se (Ia Hlop and Ia Ko communes), Duc Co (Ia Dom, Ia Nan and Ia Pnon communes), Ia Grai (Ia Chia and Ia O communes), Kong Chro (So Ro commune), and Krong Pa (Chu RCam commune) (Fig. 1). Subjects residing and/or working in these areas include both military personnel and civilians, who regularly receive primary health care at military medical facilities. Chu Pong, Duc Co and Ia Grai districts are located close to the border with Cambodia, where individuals are mainly responsible of border security (Fig. 1). Chu Se and Chu Puh are located in the centre of the province, with a predominance of soldiers working in farming. Finally, Kong Chro and Krong Pa districts are in the east, with predominance of local civilian population.

### Sample size

The asymptomatic malaria prevalence was assumed to be greater than 1.25% based on a malaria survey conducted in Krong Pa district in April 2016, in which three malaria cases were detected by blood film microscopy out of 240 people screened (Dr. Ro Mah Huan, Center for Disease Control and Prevention, Gia Lai province, unpublished data). It was determined that a minimum of 3,052 participants would be required to detect a PCR positive rate of ≥ 1.25% with a two-sided 95% confidence interval no wider than 1% with alpha and beta errors of 0.05 and 0.20, respectively. Allowing for 7% to 8% of subjects withdrawing their consent to participate in the study, it was decided to recruit at least 3,265 to 3,296 participants.

### Volunteer recruitment and enrolment

Both asymptomatic civilians and government workers from the Vietnam People’s Army eligible for medical care in the area covered by the selected military medical facilities were non-randomly invited to participate in the study. The inclusion criteria for the study were: (1) working or residing in Gia Lai province during the 14 days prior to the study visit, (2) 5 to 65 years of age, and (3) willingness to provide information (*e.g*. occupation, recent travel history, health check) and a finger prick blood sample. The exclusion criteria were: (1) being unwilling to provide consent, information or finger prick capillary blood sample, (2) inability to communicate well with the study staff, and (3) presenting with tympanic body temperature > 38 °C or reporting other malaria symptoms such as headache; these individuals were referred to the nearest health care facility. Following informed consent, study staff collected finger prick capillary blood samples for RDT (SD Bioline Malaria Ag Pf/Pv, Standard Diagnostics Inc., Abbott, Princeton, NJ, USA) and four filter paper blood spot samples were collected on 3MM filter paper (Whatman, Buckinghamshire, UK) for PCR analysis and antibody detection. Blood spots were stored individually in zip-closure plastic bags with desiccant (10 silica gel beads). Body temperature was measured, and demographic information was collected.

### Detection of malaria by PCR

DNA was extracted from filter paper blood spots (approximately 10 µL of whole blood) using the QIAamp DNA mini kit (Qiagen, Hilden, Germany) as per manufacturer’s protocol and eluted in 150 μL of AE buffer. Five µL was used as template in a single round multiplex PCR targeting small subunit (SSU) ribosomal RNA for simultaneous detection of *P. falciparum*, *P. vivax*, *P. ovale*, and *P. malariae*, as previously described [21]. PCR products were analysed by gel electrophoresis on 3% Tris–Acetate EDTA gel and compared to those produced by positive controls. Sensitivity of this method for detection of *P. falciparum* was tested using standards prepared from *P. falciparum* D6 laboratory line (Sierra-Leone, Africa). Briefly, highly synchronous ring-stage parasite cultures at 5% parasitaemia were centrifuged, washed twice in 100% plasma and resuspended at 50% haematocrit. These suspensions were dispensed into 50 μL aliquots in LoBind Eppendorf tubes and stored at -80℃. The parasite DNA was extracted from 50 μL aliquots and serially diluted. Using these standards, the lower limit of detection (LOD) for PCR was ≥ 0.25 parasites/µL. The PCR performance acceptance threshold was set tenfold greater than LOD at ≥ 2.5 parasites/µL (equivalent to 4.16 parasites in a PCR reaction or 0.833 parasites/µL DNA eluate) with respective positive DNA controls included into every batch of PCR analysis. As DNA extraction efficiency control, filter paper spots containing 50 parasites per spot were prepared using the synchronous ring-stage D6 parasites and included in every batch of DNA extraction and subsequent PCR. When testing field samples, positive PCR results were accepted (*i.e.* bands on the gel of the size corresponding to the respective positive controls) if they were reproduced in at least 2 out of 3 PCR conducted. In addition, detection of *P. knowlesi* was carried out as previously described by Imwong et al. [22].

### Molecular markers of drug resistance

Prevalence of artemisinin-resistance validated mutations in the *P. falciparum kelch-13* gene (WHO report on artemisinin and ACT resistance, August 2018, [23]; *i.e.* F446I, N458Y, M476I, Y493H, R539T, I543T, P553L, R561H and C580Y) were evaluated by sequencing of the 850 bp *Pfkelch*-13 gene region covering amino acid 427 to 687, using a previously described method [24]. For markers of piperaquine resistance, mutation in *exo-E415G* (exonuclease gene PF3D7_1362500) and copy number of *plasmepsin 2* and *3* were analysed as previously described [25, 26]. In addition, mutations in the *P. falciparum* chloroquine resistance transporter (*Pfcrt*) gene at codons 72 to 76 associated with chloroquine resistance were analysed by PCR amplification and sequencing, as previously described [27]. Copy number of *P. falciparum* multidrug resistance protein 1 (*Pfmdr1*) gene, which is associated with lumefantrine and mefloquine resistance, was determined by qPCR using SYBR Green chemistry [28]. Copy numbers > 1.5 was considered a gene duplication.

### Serological tests

Antibodies against *P. falciparum* and *P. vivax* were detected by indirect enzyme-linked immunosorbent assay (ELISA). For antibody elution, two 5 mm discs were punched from dried blood spots, placed in a 96-well plate, and mixed with 1,120 μL of elution buffer containing PBS/0.05% Tween20/0.05% NaN_3_. Plates were incubated overnight at ambient temperature (~ 23 °C) and with shaking (200 rpm), and pink/red colored eluates were stored at 4 ºC. The following recombinant proteins were produced: PfAMA-1 (3D7 strain), PfMSP1 (19 kD C-terminal fragment, 3D7), PfCSP (3D7), PvAMA-1 (Palo Alto), PvMSP1 (19 kD C-terminal fragment), PvCSP allelic variant 210 (Sal-1) and PvCSP allelic variant 247 (PNG_M69059.1). All proteins were produced at Burnet Institute (Melbourne, Australia), except PfMSP1 and PvMSP1 produced at Dalian Institute of Biotechnology (Dalian, China).

ELISA was then used to measure IgG recognition of recombinant proteins. Briefly, 96-well microplates (Corning, Corning, NY, USA) were coated with 25 ng/well of antigen in PBS or carbonate buffer, sealed and incubated overnight at 4 °C. Total IgG from human serum (Sigma-Aldrich, St. Louis, MS, USA) were used as plate standards and directly coated in control wells at three different dilutions (50 ng, 25 ng and 12.5 ng). Plates were washed and blocked with 1% skimmed milk powder in PBS/0.05% Tween20 for 2 h. Fifty μL of antibody eluates were tested in duplicate at 1/200 dilution together with positive (confirmed malaria infected patients by light microscopy) and a negative control (unexposed Vietnamese). After incubation at room temperature for 2 h, peroxidase-conjugated goat anti-human IgG secondary antibody (Sigma-Aldrich) was added for 1 h, washed and mixed with 50 µL of 2,2’-azino-di-(3-ethylbenzthiazoline sulfonic acid (Sigma-Aldrich). Reaction was stopped after 15 min with 50 μL of 2 M H_2_SO_4_, and optical density (OD) values read at 405 nm in a Multiskan™ FC Microplate Photometer (ThermoFisher, Waltham, MA, USA). Duplicates with > 25% variation were repeated once, and those with > 25% variation after repetition were excluded from analysis. The mean OD from blank wells was subtracted from samples, controls and IgG standards at each plate, and total IgG standards were then used to normalize mean sample OD between plates. Fifteen percent of the study samples (n = 575) were retested for all seven antigens to assess reproducibility of serological assays, resulting in pairwise Pearson’s correlation coefficients ranging 0.877–0.952 (all < 5% Bonferroni-adjusted significance level, Additional file 1: Figure S1). The cut-off for ELISA seropositivity against each antigen was defined as the mean OD of negative controls plus three times the standard deviation.

### Definitions and statistical analysis

The main dependent variable used this study was malaria infection based on PCR analysis. Independent demographic variables included age (stratified in seven age groups), ethnicity (Kinh versus ethnic minorities), sex, occupation (farmer, student, and government worker) and district.

Individuals were considered exposed to *P. falciparum* or *P. vivax* if they tested seropositive for at least one of the species antigens. Age-seroprevalence curves were fitted using reverse catalytic conversion models with maximum likelihood methods in R (R Core Team, v3.5.1), as previously described [17]. The model calculates one seroconversion rate (λ, *i.e.* the rate at which seronegative individuals become seropositive per year) and one seroreversion rate (ρ, i.e. rate at which seropositive individuals become seronegative per year). Seroconversion rate can thus be used as a measure of malaria force of infection or transmission intensity, whereas seroreversion rates can be interpreted as a measure of persistence of the antibody response [11]. Differences in demographic characteristics and malaria prevalence by PCR or serological data were determined using Pearson’s chi-squared (χ^2^) tests with Bonferroni corrections, or Fisher’s exact test when sample size was small. Risk factor analysis for *P. falciparum* and *P. vivax* seropositivity was conducted by running univariate logistic regression models for age, sex, ethnic group, occupation, district of residence and PCR positivity. Statistical analyses were performed in IBM SPSS 22.0 or Stata Version 12.0 (StataCorp, TX, USA), and graphs were created in Prism v8.0 (GraphPad, CA, USA). A significance level of *p* < 0.05 was defined for all tests.

## Results

### Characteristics of the study population

A total of 3,283 individuals consented to participate and were enrolled in the study from health facilities in Chu Prong (n = 330), Chu Puh (n = 600), Chu Se (n = 400), Duc Co n = 530), Ia Grai (n = 220), Kong Chro (n = 600) and Krong Pa (n = 603) districts. Overall, the participants were predominantly adults (≥ 18 years of age; 88.7%, 2911/3283), male (65.9%, 2165/3283), and belonging to an ethnic minority group (72.2%, 2371/3283), with most participants engaged in farming and outdoor work activities (90.2%, 2960/3283) (Table 1). However, demographic characteristics between districts were heterogeneous: participants enrolled in Chu Puh and Chu Se districts were exclusively adult male government workers engaged in production labor in rubber and coffee farms (n = 1000), whereas participants from Kong Chro and Krong Pa were almost exclusively from local ethnic minority groups with a high proportion of individuals under 20 years of age (≥ 30.9%; other districts: ≤ 22.3%).

**Table 1:** Study population demographic characteristics at the seven districts in Gia Lai province, Vietnam

	Total		By district														*p*-value*
			Chu Puh		Chu Se		Chu Prong		Duc Co		Ia Grai		Krong Pa		Kong Chro		
	**N**	***%***	**n**	**%**	**n**	**%**	**n**	**%**	**n**	**%**	**n**	**%**	**n**	**%**	**n**	**%**	
Participants	3283	100	600	18.3	400	12.2	330	10.1	530	16.1	220	6.7	603	18.4	600	18.3	< 0.001
*Age group*																	
5–9y	244	6.8	0	0.0	0	0.0	35	10.6	30	5.7	20	9.1	65	10.8	74	12.3	0.903
10–19y	528	16.1	134	22.3	44	11.0	18	5.5	52	9.8	10	4.5	121	20.1	149	24.8	0.057
20–29y	1381	42.1	411	68.5	354	88.5	76	23.0	212	40.0	55	25.0	133	22.1	140	23.3	< 0.001
30–39y	475	14.5	45	7.5	1	0.3	84	25.5	75	14.2	38	17.3	120	19.9	112	18.7	0.258
40–49y	287	8.7	10	1.7	1	0.3	50	15.2	60	11.3	48	21.8	65	10.8	53	8.8	0.412
50–59y	211	6.4	0	0.0	0	0.0	27	8.2	69	13.0	22	10.0	59	9.8	34	5.7	0.829
60–65y	177	5.4	0	0.0	0	0.0	40	12.1	32	6.0	27	12.3	40	6.6	38	6.3	0.765
*Sex*																	
Male	2165	65.9	599	99.8	400	100.0	190	57.6	346	65.3	113	51.4	221	36.7	296	49.3	< 0.001
Female	1118	34.1	1	0.2	0	0.0	140	42.4	184	34.7	107	48.6	382	63.3	304	50.7	
*Ethnicity*																	
Kinh	912	27.8	303	50.5	201	50.3	162	49.1	197	37.2	19	8.6	1	0.2	29	4.8	< 0.001
Ethnic minority	2371	72.2	297	49.5	199	49.8	168	50.9	333	62.8	201	91.4	602	99.8	571	95.2	
*Occupation*																	
Farmer	1737	52.9	0	0.0	0	0.0	197	59.7	393	74.2	181	82.3	475	78.8	491	81.8	< 0.001
Student	323	9.8	0	0.0	0	0.0	37	11.2	33	6.2	21	9.5	123	20.4	109	18.2	0.227
Gov. worker	1223	37.3	600	100.0	400	100.0	96	29.1	104	19.6	18	8.2	5	0.8	0	0.0	< 0.001

^*^Chi square test

The annual number of clinical malaria cases reported by the 14 communal health stations by microscopy and/or RDT between 2014 and 2017 are presented in Table 2 (detailed data for each district are presented in the Additional file 1: Table S1). Malaria prevalence (all species) declined from 0.77% (626/81,346) in 2014 to 0.12% (108/90,395) in 2016, but increased to 0.22% (201/93,184) in 2017 [29]. During this period, the average proportion of cases attributable to *P. falciparum* was 58.8% (range 38.9–73.6%), whereas *P. vivax* accounted for 39.7% (24.9–59.2%).

**Table 2:** Prevalence of clinical malaria reported by communal health stations for the seven districts in Gia Lai province, Vietnam, 2014 to 2017

Year	Total			Prevalence by district (%)						
	Population	n	%	Chu Puh	Chu Se	Chu Prong	Duc Co	Ia Grai	Krong Pa	Kong Chro
*a) Population prevalence of clinical malaria*										
2014	81,346	626	0.77	0.09	0.07	0.53	0.73	0.55	3.94	3.78
2015	85,114	300	0.35	0.04	0.02	0.28	0.36	0.10	2.08	1.72
2016	90,395	108	0.12	0.02	0.00	0.08	0.14	0.02	0.45	0.98
2017	93,184	201	0.22	0.03	0.00	0.31	0.58	0.27	0.27	0.18
*b) Population prevalence of P. falciparum cases*										
2014	81,346	404	0.50	0.06	0.03	0.15	0.47	0.34	2.44	2.88
2015	85,114	174	0.20	0.03	0.02	0.23	0.08	0.06	1.33	1.24
2016	90,395	42	0.05	0.02	0.00	0.04	0.02	0.00	0.18	0.51
2017	93,184	148	0.16	0.01	0.00	0.20	0.49	0.14	0.16	0.10
*c) Population prevalence of P. vivax cases*										
2014	81,346	214	0.26	0.02	0.04	0.38	0.26	0.20	1.46	0.79
2015	85,114	122	0.14	0.01	0.00	0.05	0.29	0.03	0.72	0.45
2016	90,395	64	0.07	0.00	0.00	0.04	0.13	0.02	0.27	0.44
2017	93,184	50	0.05	0.01	0.00	0.11	0.08	0.13	0.10	0.03

Data provided by Center for Disease Control and Prevention for Gia Lai province, Vietnam [29]

Clinical malaria was defined as symptomatic cases reported by communal health stations with a confirmed microscopy and/or RDT result

### Prevalence of asymptomatic malaria

Two individuals tested positive for malaria by RDT (0.06%, 2/3283). The prevalence of asymptomatic malaria detected by PCR was 1.74% (57/3283; *P. falciparum* 1.07%, *P. vivax* 0.40%, *P. malariae* 0.15%, and mixed infections 0.12%; Table 3). Excluding Chu Puh and Chu Se, which had a different demographic composition and little to no clinical malaria cases, the prevalence of asymptomatic malaria for the other five districts was 2.1% (49/2283). Asymptomatic individuals were detected in all districts except Chu Se district (Table 3). *P. falciparum* was the predominant species (61.4%, 35/57) followed by *P. vivax* (22.8%, 13/57). The difference in total and individual *Plasmodium* species malaria distribution was significantly different between the seven districts surveyed. *P. ovale* and *P. knowlesi* malaria were not detected in the participants’ blood samples. Out of the 57 PCR positive cases, three presented with clinical malaria within a period of two years after recruitment, including one case of acute vivax malaria seven months after recruitment (personal communication, NVT).

**Table 3:** Prevalence of malaria and Plasmodium species by PCR in the study area, stratified by seven districts in Gia Lai province, Vietnam

	Total	By district							*p*-value*
		Chu Puh	Chu Se	Chu Prong	Duc Co	Ia Grai	Krong Pa	Kong Chro	
Samples tested	3,283	600	400	330	530	220	603	600	
*PCR result, n (%)*									
Negative	3,226 (98.3)	592 (98.7)	400 (100)	327 (99.1)	506 (95.5)	219 (99.6)	588 (97.5)	594 (99)	p < 0.001
Positive	57 (1.7)	8 (1.3)	0 (0)	3 (0.9)	24 (4.5)	1 (0.5)	15 (2.5)	6 (1)	
*Species, n (%)*									
*Pf*	35 (61.4)	5 (62.5)	–	–	17 (70.8)	1 (100)	8 (53.3)	4 (66.7)	p = 0.002
*Pv*	13 (22.8)	2 (25)	–	3 (100)	7 (29.2)	–	–	1 (16.7)	
*Pm*	5 (8.8)	–	–	–	–		4 (26.7)	1 (16.7)	
*Pf* & *Pv*	1 (1.8)	1 (12.5)	–	–	–	–	–	–	
*Pf* & *Pm*	3 (5.3)	–	–	–	–	–	3 (20)	–	

*P. falciparum* – Pf; *P. vivax* – Pv; *P. malariae* – Pm; *P. falciparum* and *P. vivax—*Pf & Pv; *P. falciparum* and *P. malariae*—Pf & Pm. * Fisher’s exact test

By occupation, PCR positive samples were detected in 2.0% (35/1737) of farmers, 3.1% (10/323) of students and 1.0% (12/1223) of government workers (Additional file 1: Table S2, Fisher's exact *p* = 0.156). No significant differences in PCR malaria prevalence were seen by age group (Additional file 1: Table S3, Fisher's exact *p* = 0.523), sex (Additional file 1: Table S4, Fisher's exact *p* = 0.312) or ethnicity (Additional file 1: Table S5, Fisher's exact *p* = 0.803).

### Molecular markers of drug resistance for *P. falciparum* malaria

Molecular markers of drug resistance were analysed in samples with sufficient amount of DNA for PCR amplification and sequencing. No samples had mutations associated with artemisinin resistance in *PfKelch*-13 (0/13). Among markers associated with piperaquine resistance, none of the samples had the *exo-E415G* mutation (0/11), but 13.3% (2/15) had increased copy numbers of *plasmepsin 2/3*. Chloroquine resistant haplotype CVIET at positions 72–76 of *Pfcrt* gene was observed in 27.3% (6/22) samples. Four out of 15 samples (26.7%) had multiple copies of *Pfmdr1.* The two isolates with multiple *plasmepsin 2/3* copies also had multiple copies of *Pfmdr1* gene (2/15, 13.3%).

### Serological findings

**Fig. 2:**
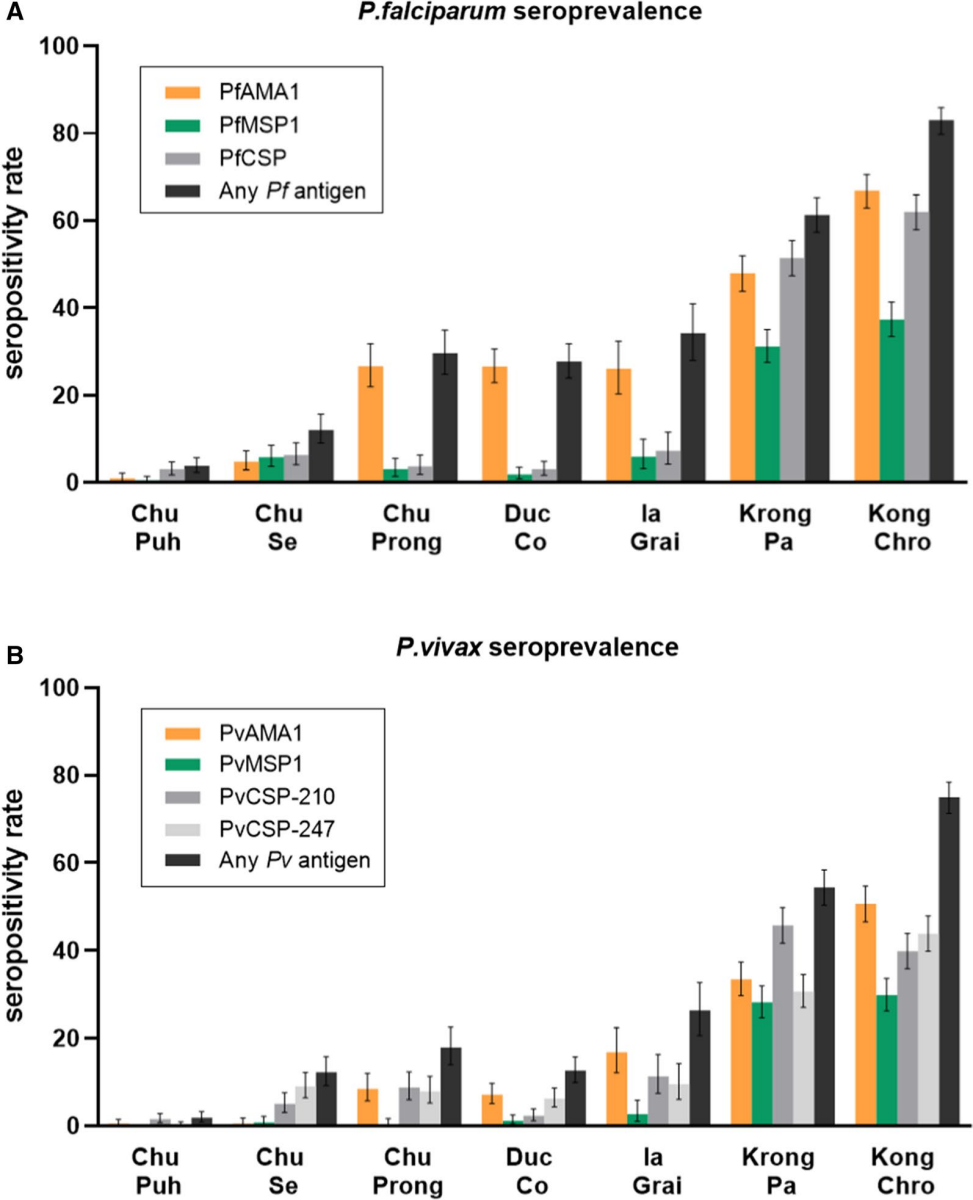
Seroprevalence of participants in seven districts in Gia Lai province against *P. falciparum* and *P. vivax* antigens. Error bars indicate 95% CI

Overall, 38.5% (1257/3262) of participants had antibodies to at least one *P. falciparum* antigen, and 31.1% (1022/3282) responded to at least one *P. vivax* antigen (Fig. 2 and Additional file 1: Table S6). The highest seroprevalence for individual antigens of each species was 30.6% (1001/3274) for PfAMA1 and 18.7% (614/3283) for PvAMA1. In contrast, the lowest seroprevalence was found against PfMSP1 (14.4%, 471/3277) and PvMSP1 (11.1%, 365/3283), respectively. Seroprevalence varied significantly by district (Additional file 1: Table S6; Pearson’s Chi-Square test *p* < 0.001) and were highest in Krong Pa and Krong Chro districts and lowest in Chu Puh and Chu Se. Noted, that participants from these latter two districts were predominately male government workers with low clinical malaria rates as well as evidence of low malaria exposure: seroprevalence in Chu Puh and Chu Se was 1% (6/600) and 4.8% (19/400) for PfAMA1, and 0.5% (3/600 and 2/400) for PvAMA1 (Additional file 1: Table S6). Due to the age and gender homogeneity these two districts were excluded from subsequent analysis.

Factors associated with *P. falciparum* and *P. vivax* exposure (defined as being seropositive to any antigen for each species) were determined using logistic regression in five of the study districts (Table 4). In these areas, 52% of the participants had evidence of previous exposure to *P. falciparum* (1178/2279) and 42% (962/2282) to *P. vivax.* There was no association between PCR positivity (*P. falciparum*: OR 0.88 [95% CI 0.50–1.55], *p* = 0.660; *P. vivax*: OR 0.72 [95% CI 0.40–1.31], *p* = 0.287) and antibody responses. Female sex, being from an ethnic minority group, farming or residing in Krong Pa and Kong Chro districts indicated an increased risk of both *P. falciparum* and *P. vivax* exposure (*p* < 0.001). Overall, there was an increased risk of seropositivity for older age groups as compared to young children, especially for *P. falciparum.*

**Table 4:** Factors associated with *P. falciparum* and *P. vivax* seropositivity to any antigen in the study area, Gia Lai province (excluding Chu Puh and Chu Se districts)

Variable	Any *P. falciparum* antigen				Any *P. vivax antigen*			
	Seropositivity		OR (95% CI)	p-value*	Seropositivity		OR (95% CI)	p-value*
	n/N	Rate (%)			n/N	Rate (%)		
*PCR*								
Negative	1163/2230	52.1			945/2233	42.3		
Positive	24/49	48.9	0.88 (0.5–1.55)	0.660	17/49	34.7	0.72 (0.40–1.31)	0.287
*Age group*								
5–9y	90/223	40.4			77/224	34.4		
10–19y	11/20	55.0	1.81 (0.72–4.54)	0.208	12/20	60.0	2.86 (1.12–7.3)	0.028
20–29y	31/116	26.7	0.54 (0.33–0.88)	0.014	26/116	22.4	0.55 (0.33–0.92)	0.024
30–39y	46/75	61.3	2.34 (1.37–4.01)	0.002	38/74	51.3	2.01 (1.18–3.43)	0.010
40–49y	38/62	61.3	2.34 (1.31–4.17)	0.004	29/62	46.8	1.68 (0.94–2.97)	0.075
50–59y	17/34	50.0	1.48 (0.72–3.04)	0.290	15/34	44.1	1.51 (0.73–3.13)	0.271
60–69y	109/176	61.9	2.4 (1.6–3.61)	< 0.001	88/177	49.7	1.89 (1.26–2.83)	0.002
*Sex*								
Male	529/1164	45.4			436/1166	37.4		
Female	658/1115	59.0	1.73 (1.46–2.04)	< 0.001	526/1116	47.1	1.49 (1.26–1.73)	< 0.001
*Ethinicity*								
Kinh	91/408	22.3			67/408	16.4		
Minority	1096/1871	58.6	4.93 (3.83–6.33)	< 0.001	895/1874	47.8	4.65 (3.53–6.14)	< 0.001
*District*								
Chu Prong	97/327	29.7			59/329	17.9		
Duc Co	147/530	27.7	0.91 (0.67–1.23)	0.544	67/530	12.6	0.66 (0.45–0.97)	0.034
Ia Grai	75/219	34.2	1.23 (0.86–1.78)	0.259	58/220	26.4	1.64 (1.09–2.47)	0.019
Krong Pa	370/603	61.4	3.76 (2.82–5.02)	< 0.001	328/603	54.4	5.46 (3.95–7.54)	< 0.001
Kong Chro	498/600	83.0	11.58 (8.42–15.92)	< 0.001	450/600	75.0	13.73 (9.8–19.22)	< 0.001
*Occupation*								
Farmer	1015/1734	58.5			798/1736	46.0		
School age	142/322	44.1	0.56 (0.44–0.71)	< 0.001	138/323	42.7	0.88 (0.69–1.11)	0.283
Worker/Soldier	30/223	13.5	0.11 (0.07–0.16)	< 0.001	26/223	11.7	0.16 (0.1–0.24)	< 0.001

^*^Univariate logistic regression

**Fig. 3:**
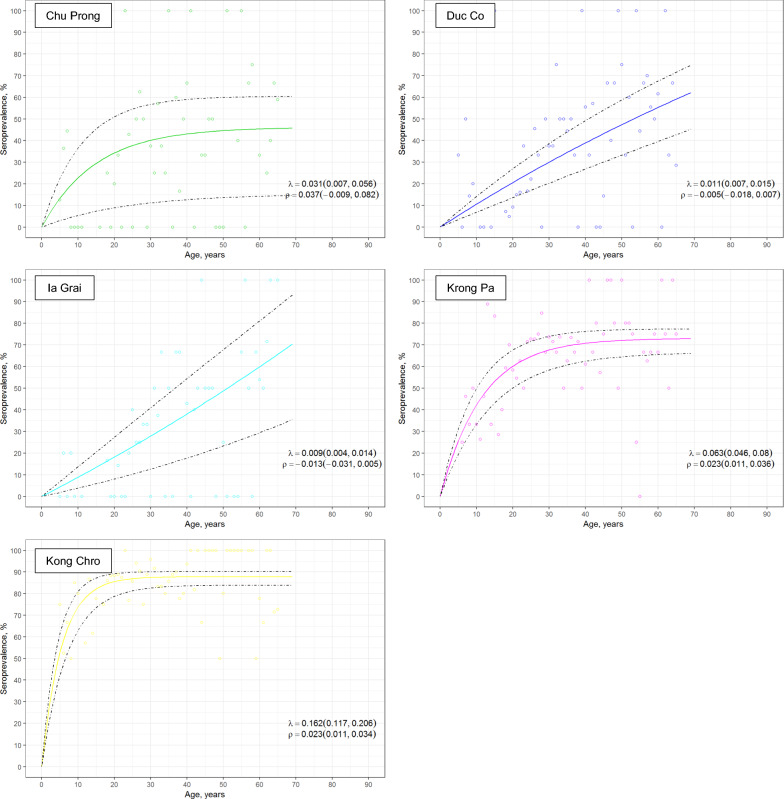
Age-seroprevalence curves for *P. falciparum* exposure across five districts in Gia Lai province, Vietnam. Exposure is defined as seropositivity to at least one *P. falciparum* antigen. Reversible catalytic conversion models allowing one seroconversion rate (λ) were fit to the data (dashed line indicates 95% CI)

**Fig. 4:**
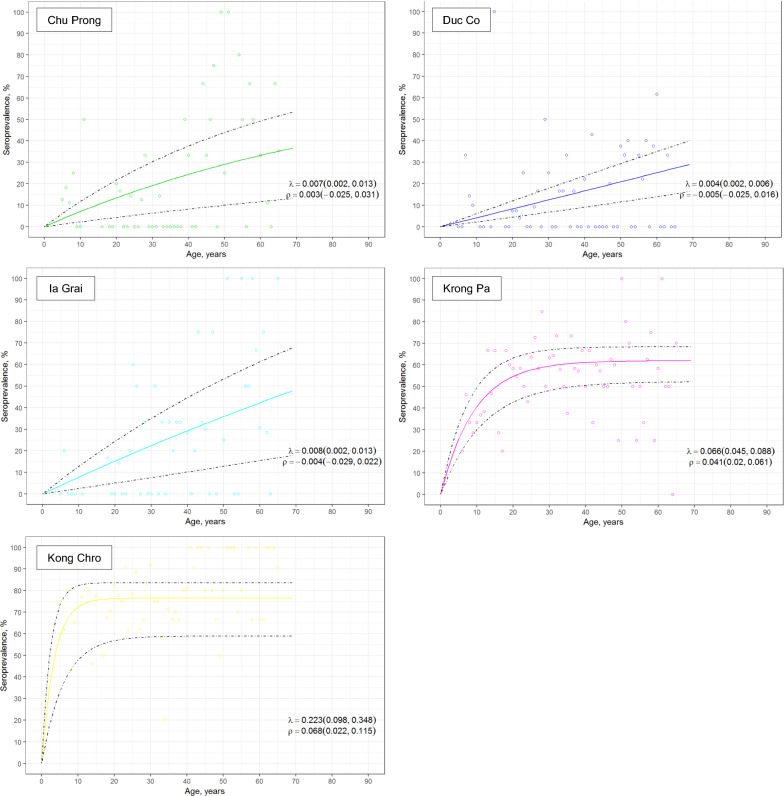
Age-seroprevalence curves for *P. vivax* exposure across five districts in Gia Lai province, Vietnam. Exposure is defined as seropositivity to at least one *P. vivax* antigen. Reversible catalytic conversion models allowing one seroconversion rate (λ) were fit to the data (dashed line indicates 95% CI)

Seroprevalence data were used to estimate malaria transmission intensity by fitting age-seroprevalence curves for *P. falciparum* and *P. vivax* exposure at each district (Figs. 3 and 4), as well as for response to individual antigens (Additional file 1: Figure S2 for *P. falciparum* antigens and Additional file 1: Figure S3 for *P. vivax* antigens). Seroconversion rates were highest in Krong Pa and Kong Chro (λ range for *P. falciparum* = 0.063–0.162; λ range for *P. vivax* = 0.066–0.223), the districts with the highest prevalence of clinical malaria in 2014–2015 (Figs. 3 and 4, Additional file 1: Table S1 and Additional file 1: Table S7). In contrast, seroconversion was lower in Chu Prong, Duc Co and Ia Grai districts (λ range for *P. falciparum* = 0.009–0.031; λ range for *P. vivax* = 0.004–0.008; Additional file 1: Table S7). In these three districts, responses elicited against PfAMA1 and PvAMA1 were the main contributors to overall exposure data, as the number of individuals seropositive for MSP1 and/or CSP antigens was low (Additional file 1: Figure S2 and Additional file 1: Figure S3).

## Discussion

Sub-clinical malaria represents a disease reservoir that should be considered when determining the malaria risk for naïve people, and for developing malaria control and elimination strategies in areas transitioning from pre-elimination to elimination. This is the first study to provide a snapshot of the burden of asymptomatic malaria in Gia Lai province, Vietnam.

The overall prevalence of asymptomatic malaria in the 3283 participants was 1.74% in December 2016–January 2017, with 96.4% (55/57) below the RDT detection threshold. This prevalence was markedly higher than the mean clinical malaria prevalence rate of 0.12% (108/90,395) reported in 2016 among residents from the 14 communes where the participants were recruited. Although the survey included a subset of the province population, other studies in similar epidemiological settings have indicated that asymptomatic *Plasmodium* carriers are substantially more prevalent than clinical cases [8, 9]. Moreover, the district selection covered geographically distinct areas, as well as people from all age groups, minority ethnicities, and different occupations. In the case of Chu Puh and Chu Se districts the recruitment included mainly adults from military forces, which could suggest a bias in the asymptomatic malaria prevalence estimates. However, both districts are semi-urban areas where a decrease in overall malaria cases has been observed in the past years and thus a lower prevalence of asymptomatic infections could be expected.

Importantly, the PCR assay in the present study using a finger prick capillary blood of approximately 10 µL and based on the PCR performance acceptance threshold was designed to detect sub-microscopic malaria infections with a sensitivity of at least 1.6 to eightfold greater than that of microscopy (the LOD in thick blood films for expert microscopists is estimated at 4 to 20 parasites/µL [30]). Moreover, we cannot exclude that the sub-microscopic reservoir was larger if using alternate molecular methods that offered greater sensitivity, such as ultra-sensitive quantitative PCR assays that use high blood volume (2–3 mL of venous blood; LOD as low as 22 parasites/mL) [9, 31], or assays targeting multi-copy gene families (LOD 30–150 parasites/mL) [32]. In this study, finger-prick capillary blood collection followed by standard PCR was selected to minimize the burden on study participants (reluctance to provide venous blood) and reduce logistic requirements and costs, both of which would have impacted on the conduct of present study as well as potential feasibilities of future surveys.

The results suggest no presence of *P. ovale* and *P. knowlesi* infections in the study population. In the present study, the PCR assay sensitivity was only tested for *P. falciparum*. Although rare, *P. ovale* infection detected by PCR has been reported in several southern Vietnam provinces (Song Be, Lam Dong, Dak Lak, and Khanh Hoa) [33] and in south-central Ninh Thuan province [34], but the species has not been reported in Gia Lai yet. Similarly, *P. knowlesi* has been reported in south-central Ninh Thuan province [35] and in central Quang Tri province [36], but not in Gia Lai. The detection of cases of asymptomatic *P. malariae* (three mixed infections of *P. falciparum* and *P. malariae*, and five mono-infections of *P. malariae*) supports other reports of cryptic or asymptomatic *P. malariae* infections in Southeast Asia [37]. The clinical and public health importance of these asymptomatic infections is unknown, but the prevalence may be sufficient to maintain transmission in the study area and warrants further investigation.

Among asymptomatic infections caused by *P. falciparum* no *Pfkelch*-13 mutations that confer artemisinin resistance were detected (0/13). This finding contrasts with other recent studies that reported a prevalence of *Pfkelch*-13 validated mutations of 74% (20/27) in Ia Grai district in 2015, and up to 89% (39/45) in Krong Pa district in 2017, in both cases C580Y mutations were predominant [19, 20]. Unlike the present study, both studies by Thanh et al. and Rovira-Vallbona et al. were clinical trials conducted with symptomatic individuals presenting with high parasite densities (> 500 parasites/μL) [19, 20]. The absence of *PfKelch-13* mutations in samples collected from asymptomatic individuals in the present study could be due to the small sample size of the subset of *P. falciparum* positive samples (13/35) for which the sequence data were obtained and/or heterogeneity in the local parasite populations. Another possible explanation is that in the absence of drug pressure resistant parasites appear to be less fit in comparison with the wild type parasites [38], and thus could be outgrown by the wild type parasites in low parasitemia asymptomatic infections. On the other hand, multiple copies of the *plasmepsin 2/3* genes and *pfmdr1* gene were found in 13.3%, (2/15) and 26.7% (4/15) of infections, respectively, including two double mutants. Amplification of *plasmepsin 2/3* confer resistance to piperaquine, the partner drug in the first-line artemisinin-combination therapy DHA-PPQ used in Vietnam up until 2020. The rate of *plasmepsin 2/3* amplification coincides with that reported in a clinical trial conducted Krong Pa district during the same period in Vietnamese subjects (10.4%, 5/48) [20]. Increases in the *pfmdr1* copy number are associated with resistance to mefloquine, which has not been used in the study area since the 1990s (personal communication with Dr. Ngo Duc Thang). This suggests the possibility of importation of resistant malaria parasite from neighboring Cambodia, where artesunate-mefloquine has been used to treat *P. falciparum* malaria in early 2000s [39]. Further investigation is needed to better understand the movement of individuals carrying malaria parasites across the international border that Gia Lai province shares with Cambodia.

This study also aimed to explore serology as an alternative malaria transmission metric for surveillance purposes. The antigens AMA-1, MSP1 and CSP, were selected as they are present in both species, represent both pre-erythrocytic and erythrocytic stage parasites and have been extensively used as markers of exposure in the past. The analysis of antibody responses of study participants found that 38.5% (1257/3262) and 31.1% (1022/3282) of individuals responded to at least one *P. falciparum* or *P. vivax* antigen, respectively. Although no other serological surveys had been conducted previously in Gia Lai, these rates compare to those found in similar epidemiological settings, such as a population survey in Quang Nam, Vietnam, in 2015 (42.3%, 145/343 for *P. falciparum* and 22.4%, 77/343 for *P. vivax*), or a study among malaria-negative adults in Oddar Meanchey, Cambodia, in 2011 (50%, 57/113 for *P. falciparum-*MSP1 and 61%, 69/113 for *P. vivax-*MSP1) [18, 40]. The relatively high seroprevalence rates as compared to parasitological estimates could be explained by (1) the fact that antibody responses reflect cumulative exposure events in the past, including asymptomatic infections, (2) the relatively long half-life (*i.e.* > 6 months) of responses against Pf/PvAMA1 or Pf/PvMSP1 antigens generated after exposure events [41, 42] and, to a minor extent, (3) missed sub-microscopic infections in the cross-sectional survey that could increase population malaria prevalence rate. Antibody decay rates (or half-lives) for the antigens used in the present study had been previously determined in a cohort from Ratanakiri province, Cambodia (bordering Gia Lai province) using multiple antibody measurements over 2 years’ time; the authors reported half-lives to be between ≈ 10 and 28 months in individuals that remained PCR negative during the follow-up (*i.e.* 297 days for PfMSP1, 569 for PfCSP, 367 for PvAMA1, 312 for PvMSP1, 885 for PvCSP210 and 725 for PvCSP247) [42]. The long half-live against CSP, particularly for the two *P. vivax* antigens, may partly explain the high seroprevalence found for CSP antigens in some of the districts included in the present study.

Age-seroprevalence curves allowed to identify two types of districts. For Chu Prong, Duc Co, and Ia Grai districts a linear association between age and seroprevalence rate was seen, suggestive of a sustained decrease in malaria prevalence in the past years. In contrast, Krong Pa and Kong Chro districts had the highest seroconversion rates for all antigens and a rapid acquisition of antibodies at early ages for both *P. falciparum* and *P. vivax* antigens, what can be interpreted as ongoing transmission of both species. Although the higher number of individuals < 20 years of age in Krong Pa and Kong Chro might have contributed to these differences, retrospective data showed these two districts had the highest clinical malaria rate among study districts in the two years preceding the survey, in line with serological results. The decrease in malaria cases reported between 2014 and 2016 in all districts by an average of 6.4-fold was not captured in the age-seroprevalence curves conducted at the end of 2016, probably due to the high immunogenicity and long duration of responses to selected antigens.

Risk factors for seropositivity were assessed after excluding the two districts with only military personnel and negligible seropositivity rates (*i.e.* Chu Puh and Chu Se). Even so, district of residence significantly increased risk of seropositivity for both *Plasmodium* species, in agreement with the higher number of malaria cases that detected in Krong Pa and Kong Chro communal health stations in recent years (Tables 2 and 4). *Plasmodium falciparum* antibody prevalence was also highest among older age groups, what may be explained by different risk behaviour compared to younger student-age groups (*i.e.* working in farming and sleeping outdoors) together with their history of exposure to infection during their lifetime. Similarly, the higher seroprevalence in females than males may be due to a higher proportion of females being farmers by occupation (83.8%; 936/1117) compared with males (68.7%; 801/1166) in these communities. Farming activities often include going far into forests where risk of malaria is higher. On the other hand, no association was seen between PCR positivity in the cross-sectional survey and *P. falciparum* or *P. vivax* seroprevalence. Although studies in low endemic areas have shown that even low-density asymptomatic infections can induce strong long-lived IgG responses against *P. vivax* [43], the number of PCR positive individuals was low. Another study using qPCR for parasite diagnosis in Quang Nam province could not identify active parasite carriers despite serological evidence of ongoing transmission in consecutive cross-sectionals [18]. As compared to parasite prevalence surveys that can only identify active infections, serological surveys in pre-elimination settings can be cost-effective tools to confirm the cease in transmission in a given area, and/or to measure changes in transmission intensity over time.

## Conclusions

Sub-clinical malaria represents a disease reservoir that should be considered when planning malaria elimination strategies and for determining the malaria risk for naïve personnel, such as travelers and military personnel visiting malaria endemic areas. In this study, the asymptomatic prevalence of malaria infections was 1.74% in seven districts of Gia Lai province, an area of low clinical malaria endemicity. The high seropositivity rates for *Plasmodium* antigens suggest that a significant proportion of participants had previous malaria exposure, and age-dependent trends allowed to differentiate districts with sustained low transmission from others with higher ongoing transmission levels of both *P. falciparum* and *P. vivax*. The results of this study can inform NMCP on the burden of asymptomatic malaria in Gia Lai and provide tools for geographical stratification of transmission, in order to design efficient public health and elimination strategies.

## Supplementary Information

**Additional file 1: Figure S1.** Reproducibility of the ELISA assays for the seven *Plasmodium* antigens for 15% of samples (n = 575). **Table S1.** Malaria prevalence for blood film and/or rapid diagnostic testing positive people in the seven districts of Gia Lai province, Vietnam. **Table S2.** PCR results by Plasmodium species and self-reported occupation of study subjects from Gia Lai province, Vietnam***.* Table S3.** PCR result and *Plasmodium* species by age-group of study subjects from Gia Lai province, Vietnam. **Table S4.** PCR result and *Plasmodium* species by sex of study subjects from Gia Lai province, Vietnam. **Table S5.** PCR result and *Plasmodium* species by ethnicity of study subjects from Gia Lai province, Vietnam. **Table S6.** Seroprevalence against *P. falciparum* and *P. vivax* by districts of study subjects from Gia Lai province, Vietnam. **Table S7.** Seroconversion rates by district*s* of study subjects from Gia Lai province, Vietnam*.* **Figure S2.** Age-seroprevalence curves for individual *P. falciparum* antigens by districts of study subjects from Gia Lai province, Vietnam. Reversible catalytic conversion models allowing one seroconversion rate (λ) were fit to the data (dashed line shows 95% CI). **Figure S3.** Age-seroprevalence curves for individual *P. vivax* antigens by districts of study subjects from Gia Lai province, Vietnam. Reversible catalytic conversion models allowing one seroconversion rate (λ) were fit to the data (dashed line shows 95% CI).

## Data Availability

The datasets generated during and/or analysed during the current study are available from the corresponding author on reasonable request.

## Abbreviations

CI: Confidence interval
DHA-PPQ: Dihydroartemisinin-piperaquine
ELISA: Enzyme-linked immunosorbent assay
GMS: Greater Mekong Subregion
ITN: Insecticide-treated net
LOD: Limit of detection
NIMPE: National Institute of Malariology, Parasitology and Entomology
NMCP: National Malaria Control Programme
OD: Optical density
OR: Odds ratio
PBS: Phosphate buffered saline
PCR: Polymerase chain reaction
PQR: Prevalence odds ratios
qPCR: Quantitative real-time PCR
RDT: Rapid diagnostic test
WHO: World Health Organization
