# Supplementary material for: Cross-sectional study of asymptomatic malaria and seroepidemiological surveillance of seven districts in Gia Lai province, Vietnam
Source: Malar J. 2022 Feb 8;21:40. doi: 10.1186/s12936-022-04060-6 (PMC8822839; doi:10.1186/s12936-022-04060-6)
Supplement: Supplementary file 1 — Additional file 1: Figure S1. Reproducibility of the ELISA assays for the seven Plasmodium antigens for 15% of samples (n = 575). Table S1. Malaria prevalence for blood film and/or rapid diagnostic testing positive people in the seven districts of Gia Lai province, Vietnam. Table S2. PCR results by Plasmodium species and self-reported occupation of study subjects from Gia Lai province, Vietnam. Table S3. PCR result and Plasmodium species by age-group of study subjects from Gia Lai province, Vietnam. Table S4. PCR result and Plasmodium species by sex of study subjects from Gia Lai province, Vietnam. Table S5. PCR result and Plasmodium species by ethnicity of study subjects from Gia Lai province, Vietnam. Table S6. Seroprevalence against P. falciparum and P. vivax by districts of study subjects from Gia Lai province, Vietnam. Table S7. Seroconversion rates by districts of study subjects from Gia Lai province, Vietnam. Figure S2. Age-seroprevalence curves for individual P. falciparum antigens by districts of study subjects from Gia Lai province, Vietnam. Reversible catalytic conversion models allowing one seroconversion rate (λ) were fit to the data (dashed line shows 95% CI). Figure S3. Age-seroprevalence curves for individual P. vivax antigens by districts of study subjects from Gia Lai province, Vietnam. Reversible catalytic conversion models allowing one seroconversion rate (λ) were fit to the data (dashed line shows 95% CI). [file 12936_2022_4060_MOESM1_ESM.docx]

**Cross-sectional study of asymptomatic malaria and seroepidemiological surveillance of seven districts in Gia Lai province, Vietnam**

Nguyen Ngoc San^1^*, Nguyen Xuan Kien^2^*, Nguyen Duc Manh^3^, Nguyen Van Thanh^3^, Marina Chavchich^4^, Nguyen Thi Huong Binh^5^, Tran Khanh Long^6^, Kimberly A. Edgel^7^, Eduard Rovira-Vallbona^6^, Michael D. Edstein^4^ and Nicholas J. Martin^7^#

^1^ Hanoi Medical University, Hanoi, Vietnam

^2^ Vietnam People’s Army Military Medical Department, Hanoi, Vietnam

^3^ Vietnam People’s Army Military Institute of Preventive Medicine, Hanoi, Vietnam

^4^ Australian Defence Force Malaria and Infectious Diseases Institute, Brisbane, Australia

^5^ National Institute of Malariology, Parasitology and Entomology, Hanoi, Vietnam

^6^ Vysnova Partners, Bethesda, MD, U.S.A

^7^ U.S. Naval Medical Research Unit TWO, Singapore, Singapore

* NNS and NXK contributed equally to this work.

#To whom correspondence should be addressed

Dr Nicholas J. Martin

U.S. Naval Medical Research Unit TWO, Singapore

Email: nicholas.j.martin10.mil@mail.mil

Additional Files

Figure S1. Reproducibility of the ELISA assays for the seven *Plasmodium* antigens for 15% of samples (n=575).

**Table S1*.*** Malaria prevalence for blood film and/or rapid diagnostic testing positive people in the seven districts of Gia Lai province, Vietnam*.*

**Table S2.** PCR results by *Plasmodium* species and self-reported occupation of study subjects from Gia Lai province, Vietnam*.*

# Table S3. PCR result and *Plasmodium* species by age-group of study subjects from Gia Lai province, Vietnam.

# Table S4. PCR result and *Plasmodium* species by sex of study subjects from Gia Lai province, Vietnam*.*

# Table S5. PCR result and *Plasmodium* species by ethnicity of study subjects from Gia Lai province, Vietnam*.*

# Table S6. Seroprevalence against *P. falciparum* and *P. vivax* by districts of study subjects from Gia Lai province, Vietnam.

**Table S7.** Seroconversion rates by district*s* of study subjects from Gia Lai province, Vietnam*.*

# Figure S2. Age-seroprevalence curves for individual *P. falciparum* antigens by districts of study subjects from Gia Lai province, Vietnam. Reversible catalytic conversion models allowing one seroconversion rate (λ) were fit to the data (dashed line shows 95% CI).

# Additional file 10: Figure S3. Age-seroprevalence curves for individual *P. vivax* antigens by districts of study subjects from Gia Lai province, Vietnam. Reversible catalytic conversion models allowing one seroconversion rate (λ) were fit to the data (dashed line shows 95% CI).

Figure S1. Reproducibility of the ELISA assays for the seven *Plasmodium* antigens for 15% of samples (n=575). Pearson’s correlation coefficients range 0.877 (PfMSP1) -0.952 (PvAMA1).

**Table S1*.*** Malaria prevalence for blood film and/or rapid diagnostic testing positive people in the seven districts of Gia Lai province, Vietnam*.*

| District and Commune | Year | Population | No. and date of participants surveyed | Malaria cases | | | | Malaria prevalence for all species (%) |
| --- | --- | --- | --- | --- | --- | --- | --- | --- |
|  |  |  |  | **Total** | **Pf** | **Total** | **Pf** | **Total** |
| Chư Prong district  Ia Mo commune | 2014 | 1972 |  | 18 | 5 | 13 | 0 | 0.91 |
|  | 2015 | 2067 |  | 9 | 9 | 0 | 0 | 0.44 |
|  | 2016 | 2120 | 220 in Dec 2016 | 1 | 1 | 0 | 0 | 0.05 |
|  | 2017 | 2354 |  | 14 | 11 | 3 | 0 | 0.59 |
|  |  |  |  |  |  |  |  |  |
| Chu Prong district  Ia Puch commune | 2014 | 1990 |  | 3 | 1 | 2 | 0 | 0.15 |
|  | 2015 | 2203 |  | 3 | 1 | 2 | 0 | 0.14 |
|  | 2016 | 2800 | 110 in Dec 2016 | 3 | 1 | 2 | 0 | 0.11 |
|  | 2017 | 3127 |  | 3 | 0 | 3 | 0 | 0.10 |
|  |  |  |  |  |  |  |  |  |
| Chu Puh district  Ia Hla commune | 2014 | 4492 |  | 10 | 7 | 3 | 0 | 0.22 |
|  | 2015 | 5074 |  | 5 | 4 | 1 | 0 | 0.10 |
|  | 2016 | 5315 | 153 in Dec 2016 | 4 | 4 | 0 | 0 | 0.08 |
|  | 2017 | 5381 | 47 in Jan 2017 | 0 | 0 | 0 | 0 | 0.00 |
|  |  |  |  |  |  |  |  |  |
| Chu Puh district  Ia Le commune | 2014 | 11408 |  | 11 | 8 | 3 | 0 | 0.10 |
|  | 2015 | 11563 |  | 3 | 2 | 1 | 0 | 0.03 |
|  | 2016 | 11782 | 200 in Dec 2016 | 1 | 1 | 0 | 0 | 0.01 |
|  | 2017 | 11601 |  | 6 | 3 | 3 | 0 | 0.05 |
|  |  |  |  |  |  |  |  |  |
| Chu Puh district  la Phang commune | 2014 | 9423 |  | 1 | 1 | 0 | 0 | 0.01 |
|  | 2015 | 9621 |  | 2 | 1 | 1 | 0 | 0.02 |
|  | 2016 | 9763 | 200 in Dec 2016 | 1 | 0 | 0 | 1 | 0.01 |
|  | 2017 | 10340 |  | 1 | 0 | 1 | 0 | 0.01 |
|  |  |  |  |  |  |  |  |  |
| Chu Se district  Ia Hlop commune | 2014 | 10046 |  | 9 | 4 | 5 | 0 | 0.09 |
|  | 2015 | 10098 |  | 3 | 3 | 0 | 0 | 0.03 |
|  | 2016 | 10114 |  | 0 | 0 | 0 | 0 | 0.00 |
|  | 2017 | 10384 | 200 in Jan 2017 | 0 | 0 | 0 | 0 | 0.00 |

*Notes: P. falciparum* – Pf; *P. vivax* – Pv; Other including mixed infections - Other

**Table S1*.*** Malaria prevalence for blood film and/or rapid diagnostic testing positive people in the seven districts of Gia Lai province, Vietnam *(*cont.*).*

| District and Commune | Year | Population | No. and date of participants surveyed | Malaria cases | | | | Malaria prevalence for all species (%) |
| --- | --- | --- | --- | --- | --- | --- | --- | --- |
|  |  |  |  | **Total** | **Pf** | **Pv** | **Other** |  |
| Chu Se district  Ia Ko commune | 2014 | 5358 |  | 2 | 1 | 1 | 0 | 0.04 |
|  | 2015 | 5696 |  | 0 | 0 | 0 | 0 | 0.00 |
|  | 2016 | 6219 |  | 0 | 0 | 0 | 0 | 0.00 |
|  | 2017 | 6313 | 200 in Jan 2017 | 0 | 0 | 0 | 0 | 0.00 |
|  |  |  |  |  |  |  |  |  |
| Đuc Co district  Ia Dom commune | 2014 | 5517 |  | 22 | 14 | 8 | 0 | 0.40 |
|  | 2015 | 6312 |  | 13 | 6 | 7 | 0 | 0.21 |
|  | 2016 | 7019 |  | 4 | 0 | 4 | 0 | 0.06 |
|  | 2017 | 7742 | 310 in Jan 2017 | 14 | 9 | 5 | 0 | 0.18 |
|  |  |  |  |  |  |  |  |  |
| Đuc Co district  Ia Nan commune | 2014 | 5179 |  | 68 | 37 | 31 | 0 | 1.31 |
|  | 2015 | 6218 |  | 42 | 5 | 37 | 0 | 0.68 |
|  | 2016 | 7757 |  | 21 | 3 | 18 | 0 | 0.27 |
|  | 2017 | 8064 | 110 in Jan 2017 | 84 | 72 | 11 | 1 | 1.04 |
|  |  |  |  |  |  |  |  |  |
| Đuc Co district  Ia Pnon commune | 2014 | 4385 |  | 20 | 20 | 0 | 0 | 0.46 |
|  | 2015 | 4497 |  | 7 | 2 | 5 | 0 | 0.16 |
|  | 2016 | 4599 | 88 in Dec 2016 | 3 | 0 | 3 | 0 | 0.07 |
|  | 2017 | 4704 | 22 in Jan 2017 | 20 | 20 | 0 | 0 | 0.43 |
|  |  |  |  |  |  |  |  |  |
| Ia Grai district  Ia Chia commune | 2014 | 5371 |  | 2 | 1 | 1 |  | 0.04 |
|  | 2015 | 5460 |  | 0 | 0 | 0 | 0 | 0.00 |
|  | 2016 | 5637 |  | 0 | 0 | 0 | 0 | 0.00 |
|  | 2017 | 5712 | 110 in Jan 2017 | 2 | 1 | 1 | 0 | 0.04 |

*Notes: P. falciparum* – Pf; *P. vivax* – Pv; Other including mixed infections - Other

**Table S1*.*** Malaria prevalence for blood film and/or rapid diagnostic testing positive people in the seven districts of Gia Lai province, Vietnam *(*cont.*).*

| District and Commune | Year | Population | No. and date of participants surveyed | Malaria cases | | | | Malaria prevalence for all species (%) |
| --- | --- | --- | --- | --- | --- | --- | --- | --- |
|  |  |  |  | **Total** | **Pf** | **Pv** | **Other** |  |
| Ia Grai district  Ia O commune | 2014 | 5901 |  | 60 | 37 | 22 | 1 | 1.02 |
|  | 2015 | 5975 |  | 12 | 7 | 4 | 1 | 0.20 |
|  | 2016 | 6711 |  | 2 | 0 | 2 | 0 | 0.03 |
|  | 2017 | 6816 | 110 in Jan 2017 | 32 | 17 | 15 | 0 | 0.47 |
|  |  |  |  |  |  |  |  |  |
| Kong Chro district  So Ro commune | 2014 | 3781 |  | 143 | 109 | 30 | 4 | 3.78 |
|  | 2015 | 3786 |  | 65 | 47 | 17 | 1 | 1.72 |
|  | 2016 | 3885 |  | 38 | 20 | 17 | 1 | 0.98 |
|  | 2017 | 3897 | 600 in Jan 2017 | 7 | 4 | 1 | 2 | 0.18 |
|  |  |  |  |  |  |  |  |  |
| Krong Pa district  Chur Cam commune | 2014 | 6523 |  | 257 | 159 | 95 | 3 | 3.94 |
|  | 2015 | 6544 |  | 136 | 87 | 47 | 2 | 2.08 |
|  | 2016 | 6674 | 561 in Dec 2016 | 30 | 12 | 18 | 0 | 0.45 |
|  | 2017 | 6749 | 42 in Jan 2017 | 18 | 11 | 7 | 0 | 0.27 |

*Notes: P. falciparum* – Pf; *P. vivax* – Pv; Other including mixed infections - Other

**Table S2.** PCR results by *Plasmodium* species and self-reported occupation of study subjects from Gia Lai province, Vietnam*.*

|  | Occupation | | | | *p-value** |
| --- | --- | --- | --- | --- | --- |
|  | **Farmer** | **Student** | **Government**  **worker** | **Total** |  |
| PCR: | n (%) | n (%) | n (%) | n (%) |  |
| negative | 1702 (98) | 313 (96.9) | 1211 (99) | 3226 (98.3) | *p* = 0.015 |
| positive | 35 (2.0) | 10 (3.1) | 12 (1.0) | 57 (1.7) |  |
| Total | 1737 | 323 | 1223 | 3283 |  |
| Species: |  | | | | |
| Pf | 24 (68.6) | 5 (50) | 6 (50) | 35 (61.4) | *p* = 0.156 |
| Pv | 5 (14.3) | 3 (30) | 5 (41.7) | 13 (22.8) |  |
| Pm | 3 (8.6) | 2 (20) | - | 5 (8.8) |  |
| Pf & Pv | - | - | 1 (8.3) | 1 (1.8) |  |
| Pf & Pm | 3 (8.6) | - | - | 3 (5.3) |  |
| Total | 35 | 10 | 12 | 57 |  |

*Notes: P. falciparum* – Pf; *P. vivax* – Pv; *P. malariae* – Pm; *P. falciparum* and *P. vivax -* Pf & Pv; *P. falciparum* and *P. malariae* - Pf & Pm; * Fisher’s exact test

# Table S3. PCR result and *Plasmodium* species by age-group of study subjects from Gia Lai province, Vietnam.

|  | Age group | | | | | | | | *p-value** |
| --- | --- | --- | --- | --- | --- | --- | --- | --- | --- |
|  | **5-9** | **10-19** | **20 – 29** | **30 – 39** | **40 – 49** | **50 – 59** | **Above 60** | **Total** |  |
| PCR: | **n (%)** | **n (%)** | **n (%)** | **n (%)** | **n (%)** | **n (%)** | **n (%)** | **n (%)** |  |
| negative | 219 (97.8) | 518 (98.1) | 1362 (98.6) | 464 (97.7) | 280 (97.6) | 207 (98.1) | 176 (99.4) | 3226 (98.3) | *p* =0.523 |
| positive | 5 (2.2) | 10 (1.9) | 19 (1.4) | 11 (2.3) | 7 (2.4) | 4 (1.9) | 1 (0.6) | 57 (1.7) |  |
| Total | 224 (100) | 528 (100) | 1381 (100) | 475 (100) | 287 (100) | 211 (100) | 177  (100) | 3283  (100) |  |
| Species: |  |  |  |  |  |  |  |  |  |
| Pf | 2 (40) | 5 (50) | 13 (68.4) | 6 (54.6) | 5 (71.4) | 3 (75) | 1 (100) | 35 (61.4) | *p* =0.818 |
| Pv | 2 (40) | 3 (30) | 4 (21.1) | 3 (27.3) | 1 (14.3) | - | - | 13 (22.8) |  |
| Pm | 1 (20) | 1 (10) | 2 (10.5) | 1 (9.1) | - | - | - | 5 (8.8) |  |
| Pf & Pv | - | 1 (10) | - | - | - | - | - | 1 (1.8) |  |
| Pf & Pm | - | - | - | 1 (9.1) | 1 (14.3) | 1 (25) | - | 3 (5.3) |  |
| Total | 5 (100) | 10 (100) | 19 (100) | 11 (100) | 7 (100) | 4 (100) | 1 (100) | 57 (100) |  |

*Notes: P. falciparum* – Pf; *P. vivax* – Pv; *P. malariae* – Pm; *P. falciparum* and *P. vivax -* Pf & Pv; *P. falciparum* and *P. malariae* - Pf & Pm; * Fisher’s exact test

# Table S4. PCR result and *Plasmodium* species by sex of study subjects from Gia Lai province, Vietnam.

|  | Sex | | | |
| --- | --- | --- | --- | --- |
|  | **male** | **female** | **Total** | ***p-value**** |
| PCR: | **n (%)** | **n (%)** | **n (%)** |  |
| negative | 2131 (98.4) | 1095 (97.9) | 3226 (98.3) | *p* = 0.312 |
| positive | 34 (1.6) | 23 (2.1) | 57 (1.7) |  |
| Total | 2165 | 1118 | 3283 |  |
| Species: |  |  |  |  |
| Pf | 21 (61.8) | 14 (60.9) | 35 (61.4) | *p* = 0.954 |
| Pv | 8 (23.5) | 5 (21.7) | 13 (22.8) |  |
| Pm | 3 (8.8) | 2 (8.7) | 5 (8.8) |  |
| Pf & Pv | 1 (2.9) | - ( - ) | 1 (1.8) |  |
| Pf & Pm | 1 (2.9) | 2 (8.7) | 3 (5.3) |  |
| Total | 34 | 23 | 57 |  |

*Notes: P. falciparum* – Pf; *P. vivax* – Pv; *P. malariae* – Pm; *P. falciparum* and *P. vivax -* Pf & Pv; *P. falciparum* and *P. malariae* - Pf & Pm; * Fisher’s exact test

# Table S5. PCR result and *Plasmodium* species by ethnicity of study subjects from Gia Lai province, Vietnam.

|  | Ethnicity | | | |
| --- | --- | --- | --- | --- |
|  | **Kinh** | **Ethnic Minority** | **Total** | ***p-value**** |
| PCR: | **n (%)** | **n (%)** | **n (%)** |  |
| negative | 897 (98.4) | 2329 (98.2) | 3226 (98.3) | *p* = 0.803 |
| positive | 15 (1.6) | 42 (1.8) | 57 (1.7) |  |
| Total | 912 | 2371 | 3283 |  |
| Species: |  |  |  |  |
| Pf | 7 (46.7) | 28 (66.7) | 35 (61.4) | *p* = 0.024 |
| Pv | 7 (46.7) | 6 (14.3) | 13 (22.8) |  |
| Pm | - | 5 (11.9) | 5 (8.8) |  |
| Pf & Pv | 1 (6.7) | - | 1 (1.8) |  |
| Pf & Pm | - | 3 (7.1) | 3 (5.3) |  |
| Total | 15 | 42 | 57 |  |

*Notes: P. falciparum* – Pf; *P. vivax* – Pv; *P. malariae* – Pm; *P. falciparum* and *P. vivax -* Pf & Pv; *P. falciparum* and *P. malariae* - Pf & Pm; * Fisher’s exact test

# Table S6. Seroprevalence against *P. falciparum* and *P. vivax* by districts of study subjects from Gia Lai province, Vietnam.

|  | **District** | | | | | | | | | | | | | | **Total** | | ***p-value**** |
| --- | --- | --- | --- | --- | --- | --- | --- | --- | --- | --- | --- | --- | --- | --- | --- | --- | --- |
|  | **Chu Puh** | | **Chu Se** | | **Chu Prong** | | **Duc Co** | | **Ia Grai** | | **Krong Pa** | | **Kong Chro** | |  |  |  |
|  | **n/N** | **%** | **n/N** | **%** | **n/N** | **%** | **n/N** | **%** | **n/N** | **%** | **n/N** | **%** | **n/N** | **%** | **n/N** | **%** |  |
| ***P. falciparum:*** |  |  |  |  |  |  |  |  |  |  |  |  |  |  |  |  |  |
| **PF AMA1** | 6/592 | 1.0 | 19/400 | 4.8 | 88/330 | 26.7 | 141/530 | 26.6 | 57/219 | 26.0 | 289/603 | 47.9 | 401/600 | 66.8 | 1001/3274 | 30.6 | <0.001 |
| **PF MSP1** | 3/596 | 0.5 | 23/399 | 5.8 | 10/329 | 3.0 | 10/530 | 1.9 | 13/220 | 5.9 | 188/603 | 31.2 | 224/600 | 37.3 | 471/3277 | 14.4 | <0.001 |
| **PF CSP** | 18/597 | 3.0 | 25/399 | 6.3 | 12/328 | 3.7 | 16/530 | 3.0 | 16/220 | 7.3 | 310/603 | 51.4 | 372/600 | 62.0 | 769/3277 | 23.5 | <0.001 |
| **Any Pf antigen** | 22/585 | 3.8 | 48/398 | 12.1 | 97/327 | 29.7 | 147/530 | 27.7 | 75/219 | 34.2 | 370/603 | 61.4 | 498/600 | 83.0 | 1257/3262 | 38.5 | <0.001 |
| ***P. vivax:*** |  |  |  |  |  |  |  |  |  |  |  |  |  |  |  |  |  |
| **PV AMA1** | 3/600 | 0.5 | 2/400 | 0.5 | 28/330 | 8.5 | 38/530 | 7.2 | 37/220 | 16.8 | 202/603 | 33.5 | 304/600 | 50.7 | 614/3283 | 18.7 | <0.001 |
| **PV MSP1** | 0/600 | 0.0 | 3/400 | 0.8 | 1/330 | 0.3 | 6/530 | 1.1 | 6/220 | 2.7 | 170/603 | 28.2 | 179/600 | 29.8 | 365/3283 | 11.1 | <0.001 |
| **PV CSP210** | 9/600 | 1.5 | 20/400 | 5.0 | 29/330 | 8.8 | 12/530 | 2.3 | 25/220 | 11.4 | 276/603 | 45.8 | 239/600 | 39.8 | 610/3283 | 18.6 | <0.001 |
| **PV CSP247** | 1/600 | 0.2 | 36/400 | 9.0 | 26/329 | 7.9 | 33/530 | 6.2 | 21/220 | 9.5 | 185/603 | 30.7 | 263/600 | 43.8 | 565/3282 | 17.2 | <0.001 |
| **Any Pv antigen** | 11/600 | 1.8 | 49/400 | 12.3 | 59/329 | 17.9 | 67/530 | 12.6 | 58/220 | 26.4 | 328/603 | 54.4 | 450/600 | 75.0 | 1022/3282 | 31.1 | <0.001 |

*Notes: P. falciparum* – Pf; *P. vivax* – Pv, * Chisquare test

**Table S7.** Seroconversion rates by districts in study subjects from Gia Lai province, Vietnam*.*

|  | District | | | | | | | | | |
| --- | --- | --- | --- | --- | --- | --- | --- | --- | --- | --- |
|  | **Chu Prong** | | **Duc Co** | | **Ia Grai** | | **Krong Pa** | | **Kong Chro** | |
|  | **λ** | **95% CI** | **λ** | **95% CI** | **λ** | **95% CI** | **λ** | **95% CI** | **λ** | **95% CI** |
| *P. falciparum:* | |  |  |  |  |  |  |  |  |  |
| PfAMA1 | 0.025 | 0.007 - 0.043 | 0.010 | 0.006 - 0.014 | 0.006 | 0.002 - 0.009 | 0.032 | 0.024 - 0.039 | 0.068 | 0.053 - 0.082 |
| PfMSP1 | 0.009 | -0.022 - 0.040 | 0.006 | -0.064 - 0.075 | 0.012 | ind. | 0.029 | 0.017 - 0.040 | 0.041 | 0.023 - 0.058 |
| PfCSP | 0.070 | -0.54 - 0.688 | 0.008 | ind. | 0.007 | -0.02 - 0.035 | 0.066 | 0.044 - 0.089 | 0.148 | 0.075 - 0.22 |
| Any Pf antigen | 0.031 | 0.007 - 0.056 | 0.011 | 0.007 - 0.015 | 0.009 | 0.004 - 0.014 | 0.063 | 0.046 - 0.080 | 0.162 | 0.117 - 0.206 |
| *P. vivax:* |  |  |  |  |  |  |  |  |  |  |
| PvAMA1 | 0.004 | 0 - 0.009 | 0.004 | 0.001 - 0.007 | 0.004 | 0.001 - 0.007 | 0.026 | 0.016 - 0.035 | 0.050 | 0.032 - 0.068 |
| PvMSP1 | ind. | ind. | 0.003 | -0.002 - 0.008 | 0.005 | ind. | 0.029 | 0.016 - 0.042 | 0.034 | 0.016 - 0.053 |
| PvCSP210 | 0.003 | 0.001 - 0.004 | 0.006 | ind*.* | 0.006 | -0.001 - 0.012 | 0.056 | 0.035 - 0.077 | 0.149 | -0.016 - 0.314 |
| PvCSP247 | 0.131 | -2.352 - 2.614 | 0.047 | -0.149 - 0.242 | 0.005 | -0.002 - 0.012 | 0.045 | 0.022 - 0.069 | 0.111 | 0.026 - 0.195 |
| Any Pv antigen | 0.007 | 0.002 - 0.013 | 0.004 | 0.002 - 0.006 | 0.008 | 0.002 - 0.013 | 0.066 | 0.045 - 0.088 | 0.223 | 0.098 - 0.348 |

*Notes: P. falciparum* – Pf; *P. vivax* – Pv; λ - rate at which seronegative individuals become seropositive per year; indeterminate - ind.

# Figure S2. Age-seroprevalence curves for individual *P. falciparum* antigens by districts in study subjects from Gia Lai province, Vietnam. Reversible catalytic conversion models allowing one seroconversion rate (λ) were fit to the data (dashed line shows 95% CI).

# Figure S2. Age-seroprevalence curves for individual *P. falciparum* antigens by districts in study subjects from Gia Lai province, Vietnam. Reversible catalytic conversion models allowing one seroconversion rate (λ) were fit to the data (dashed line shows 95% CI). *(*cont.*).*

# Figure S2. Age-seroprevalence curves for individual *P. falciparum* antigens by districts in study subjects from Gia Lai province, Vietnam. Reversible catalytic conversion models allowing one seroconversion rate (λ) were fit to the data (dashed line shows 95% CI). *(*cont.*).*

#

# Additional file 10: Figure S3. Age-seroprevalence curves for individual *P. vivax* antigens by districts in study subjects from Gia Lai province, Vietnam. Reversible catalytic conversion models allowing one seroconversion rate (λ) were fit to the data (dashed line shows 95% CI).

# Additional file 10: Figure S3. Age-seroprevalence curves for individual *P. vivax* antigens by districts in study subjects from Gia Lai province, Vietnam. Reversible catalytic conversion models allowing one seroconversion rate (λ) were fit to the data (dashed line shows 95% CI). *(*cont.*).*

# Additional file 10: Figure S3. Age-seroprevalence curves for individual *P. vivax* antigens by districts in study subjects from Gia Lai province, Vietnam. Reversible catalytic conversion models allowing one seroconversion rate (λ) were fit to the data (dashed line shows 95% CI). *(*cont.*).*

# Additional file 10: Figure S3. Age-seroprevalence curves for individual *P. vivax* antigens by districts in study subjects from Gia Lai province, Vietnam. Reversible catalytic conversion models allowing one seroconversion rate (λ) were fit to the data (dashed line shows 95% CI). *(*cont.*).*
